# Preparation of PVA/waste oyster shell powder composite as an efficient adsorbent of heavy metals from wastewater

**DOI:** 10.1016/j.heliyon.2022.e11938

**Published:** 2022-11-29

**Authors:** Zhenfeng Zhou, Yinuo Wang, Shu Sun, Yicheng Wang, Liang Xu

**Affiliations:** School of Resources and Environment, Qingdao Agricultural University, Qingdao 266109, China

**Keywords:** Poly (vinyl alcohol), Oyster shell powder, Heavy metals, Adsorption, Modification

## Abstract

The accumulation of discarded mollusk shells has occupied a large land area and caused severe environmental pollution problems. Discarded mollusk shells are mainly composed of calcium carbonate and therefore can be used for the removal of heavy metals from the contaminated aquatic environment. Theoretically, shells with a smaller powder size have a higher adsorption capacity for heavy metal ions. However, the agglomeration and the outflow of small particles limit the applications of mollusk shells in water treatment practices. To overcome the shortcomings of mollusk shells in heavy metals adsorptions, a polymer composite material comprising poly (vinyl alcohol)/oyster shell powder (PVA-OSP) was prepared with the solution casting method for the adsorption of heavy metal ions from wastewater. The structures and the heavy metal adsorption properties of the oyster shell powder (OSP) and the composite PVA-OSP were studied and compared. Analysis results of XRD and FT-IR showed a successful combination of OSP and PVA by a chemical cross-linking modified with sodium silicate. The composite PVA-OSP has good thermal stability for common adsorption processes. The adsorption results showed that the adsorption capacity of the PVA-OSP composite for both Cu^2+^ and Cd^2+^ was much higher than that of the OSP. The adsorptions of Cu^2+^ and Cd^2+^ on the OSP followed the pseudo-second-order kinetic model as well as the Temkin and Freundlich isotherm model. Comparatively, the adsorptions of heavy metal cations on the PVA-OSP followed the pseudo-first-order kinetic model as well as the Temkin and Langmuir isotherm model. In conclusion, this study showed that the PVA-OSP composite materials may be useful in the treatment of wastewater polluted by heavy metals.

## Highlights

• Modified shell composite overcomes shortcomings of oyster shell as an adsorbent.
• Modified shell composite shows better removal efficiency for heavy metal pollution.
• Combination of organic and inorganic matters may result in improvement in adsorption.
• The reaction conditions for adsorbent composite production are mild.

## Graphical abstract

**Figure:**
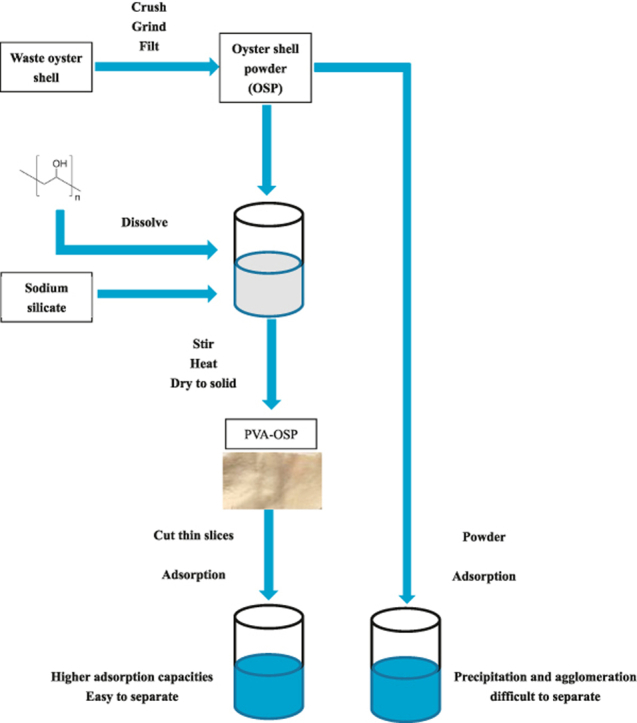

## 1 Introduction

Due to industrialization, the rapid growth of the world population, and the intensification of agriculture, the water pollution caused by heavy metals is increasing on daily basis. Fossil fuel combustion, mining, smelting, material corrosion, waste disposal, and agricultural activities are the main anthropogenic sources of heavy metal pollution (Hashemian et al., 2015; Ngah et al., 2011; Tamjidi and Ameri, 2020; Zhong et al., 2021). Heavy metals are highly toxic, not easy to be metabolized, and easy to accumulate in the environment and the organisms. The accumulation and enrichment of heavy metals often lead to the degradation of water quality and the imbalance of ecosystems (Tamjidi and Ameri, 2020). At present, the commonly used methods for treating heavy metal wastewater include chemical precipitation, flotation, solvent extraction, cementation onto iron, membrane filtration, ion exchange, electrolysis, bioremediation, and adsorption (Xu et al., 2019; Zhong et al., 2021).

The adsorption methods are simple, easy to operate, and have good treatment results (Jinendra et al., 2019a; Jinendra et al., 2019b; Xia et al., 2021). The selection of adsorbents, however, is a crucial step in the successful removal of heavy metals from wastewater by adsorption. In the selection, the adsorption effect, preparation cost, and regeneration performance of adsorbents have to be comprehensively considered (Pu et al., 2019; Homagai et al., 2022). Activated carbon is the most commonly used adsorbent, but high cost limits its wide use (Hsu, 2009; Pu et al., 2019; Tamjidi and Ameri, 2020). Therefore, it is necessary to find an alternative material for heavy metals adsorption (Liu et al., 2019).

Shellfish aquaculture is developing rapidly and the amount of waste shell generated from shellfish aquaculture is huge (Kim and Singh, 2022). Shell powder is a typical natural adsorption material with a large specific surface area, high porosity, and a low cost (depending on the proper process). The shells have strong abilities for heavy metal adsorption due to the composition of calcium carbonate and layer structures (Lu et al., 2021; Xia et al., 2021). However, the raw shell without modification generally has the characteristics of a simple surface structure, few pores, uneven particle sizes, small specific surface area, and an associated limited adsorption capacity (Pu et al., 2019; Xu et al., 2019). Modification of the shell as an adsorbent can overcome the problems of low adsorption capacity, single selectivity, and difficult solid-liquid separation to some certain extent. Through thermal, acid, or grinding treatment of shell powder, the shell structure becomes loose, the porosity and specific surface area increase and the adsorption performance could be improved. It has been shown that the modification of oyster shells by phosphoric acid made calcium carbonate change to calcium phosphate and that the modified shell exhibited stronger sorption of lead (Kim and Singh, 2022). Homogeneous porous shell particles obtained by pulverization or thermal treatment showed a good adsorption capacity to several heavy metals (Alidoust et al., 2015; Xu et al., 2019; Xia et al., 2021). To maximize the adsorption effect, the adsorbent is usually used in the form of powders to obtain a large surface area and surface energy. However, shell powder is easy to lose with water current and difficult to collect and reuse. In addition, shell powder is easy to agglomerate in water, which reduces the adsorption effect. All these problems limit the large-scale application of shells in wastewater treatment (Pu et al., 2019; Xu et al., 2019). Integrating the shell powders into polymeric materials as filler might be a method to solve these problems (Mallakpour and Khadem, 2017b; Zaharia et al., 2021; Zhang et al., 2021).

Poly(vinyl alcohol) (PVA) is a cheap, highly hydrophilic, non-toxic, and highly stable polymer with a three-dimensional network structure (Zhang and Zhang, 2020; Ge et al., 2021), which could be applied as sorbents to remove heavy metals, dyes, phosphate, and other pollutants from wastewaters (Ngah et al., 2011; Agarwal et al., 2016; Marvdashti et al., 2017). However, pure PVA alone cannot achieve a good adsorption effect. In previous studies, a new adsorbent PVA/MgO_2_ nanocomposite was designed for the selective adsorption of methylene blue (Naim and El-Shamy, 2021). PVA synthetic nanocomposite, combined with CaCO_3_ modified by dicarboxylic acid-functionalized molecules, tragacanth gum mediated by glutaraldehyde, or γ-aminopropyl triethoxy silane, exhibited a good adsorption capacity for Cd^2+^, Pb^2+,^ and Cu^2+^ (Mallakpour and Khadem, 2017a, 2017b, 2017c). A recyclable polymer composite material comprising of PVA/bacterial cellulose/CaCO_3_ was prepared and used for the adsorption of various toxic heavy metal ions (Zhang et al., 2021). The fillers used in previous studies to improve the adsorption effect of PVA on heavy metals, such as filler CaCO_3_, are mostly synthesized through chemical reactions. Zhang et al. (2021) prepared Na_2_CO_3_ solution first, and then added Na_2_CO_3_ solution to an emulsion containing PVA and CaCl_2_ under mechanical agitation to slowly generate CaCO_3_. The main component of mollusk shells is CaCO_3_. However, shells have abundant irregular porous surfaces, but CaCO_3_ produced through chemical synthesis does not have such a structure (Pu et al., 2019). As a rich source of CaCO_3_, discarded mollusk shells may be integrated into the structure of polymer materials.

To overcome the shortcomings of shells as adsorbent and realize the reasonable application of waste shells in the treatment of wastewater polluted by heavy metal, the PVA-OSP composite material was prepared by coating oyster shell powder (OSP) with PVA through Na_2_SiO_3_-mediated crosslinking for the first time. FT-IR, XRD, SEM, and TG-DSC were used to characterize the structural properties and thermal stability of the PVA-OSP composite. In addition, the adsorption properties and associated mechanisms of the composite and OSP were studied and compared by a batch adsorption experiment.

## 2 Materials and methods

### 2.1 Materials

PVA (hydrolysis degree: 88%; purity: 99%; molecular weight: 140,000 g mol^−1^), copper chloride (purity: 99%), cadmium chloride (purity: 99%), and sodium silicate (purity measured as Na_2_O, w%: 19.3–22.8%) were purchased from Sinopharm Chemical Reagent Co., Ltd., Shanghai, China. Oyster shells were obtained from Chengyang aquatic products wholesale market, Qingdao, Shandong Province, China.

### 2.2 Preparation of the OSP adsorbent and the PVA-OSP composite adsorbent

Oyster shells were first cleaned and washed with a wire brush. The cleaned oyster shells were dried in an oven at 70 °C. Shells were weighed until the difference between two consecutive times was less than 0.1%. Dried oyster shells were crushed in a high-speed universal pulverizer (FW100, Taisite Co., Ltd., Tianjin, China) to pass a 200-mesh sieve (pore size is 74 μm). The sieved powders were ground again in an airflow pulverizer (HBM-109, Hanbo Co., Ltd., Tianjin, China) to pass a filter bag of 10 microns. The oyster shell powders (OSP hereafter) were then oven-dried at 70 °C to constant weight and stored at room temperature for further use.

A certain amount of dried PVA was soaked in deionized water for 24 h, then stirred and heated at 90 °C for dissolution. After the dissolving of PVA and naturally cooling, deionized water was added to make a 5 wt% PVA solution. Quantified OSP and sodium silicate were added into the PVA solution, which was then vigorously stirred at 90 °C for 2 h. After that, the mixtures were continuously stirred at room temperature for 2 h. Then, the mixed solution and pure PVA were poured into different Teflon petri dishes and dried at 40 °C for 24 h to obtain the solid PVA-OSP composite and the pure PVA film. Thin slices were cut from the composite with a sharp blade for further characterization and adsorption experiments.

### 2.3 Characterization of adsorbents

The crystal structures data of the pure PVA, the OSP, and the PVA-OSP were collected by an X-Ray Diffractometer (TD-3700, Dandong Tongda Science & Technology Co., Ltd., China) with the scanning range 2θ = 10–80° and the scanning increment of 0.02°. The chemical structures of the samples were scanned by an FT-IR spectrometer (Nicolet isl0, Thermo, the USA), with frequencies ranging from 400 cm^−1^ to 4000 cm^−1^. Thermoanalytical characterizations of materials were performed in nitrogen flow using a Simultaneous Thermal Analyzer (SDT650, TA Instruments, the USA) at 20 °C min^−1^ heating rate from 25 °C up to 800 °C. The surface morphologies of the materials were determined using a Field Emission Scanning Electron Microscope (Gemini 500, Carl Zeiss Microscopy Deutschland GmbH, Germany).

### 2.4 Adsorption experiments

Different addition amounts of adsorbents, initial pH value, adsorption time, adsorption temperature, and initial concentration of Cu^2+^ and Cd^2+^ were set respectively to explore the static adsorption effect of adsorbents on heavy metal ions. Heavy metal ions were made in solution using CuCl_2_ and CdCl_2_, respectively.

The amount effect of adsorbents was conducted by adding adsorbents ranging from 0.02 to 1 g into 100 mL of 50 mg L^−1^ heavy metal ion solution (pH was about 7). Solutions were allowed to stand for 24 h at 30 °C in an oven. The supernatants were taken for ion concentration measurement by ICP-OES (OPTMA8000DV, PerkinElmer, Inc., the USA). The same sampling and analysis methods were used for the following adsorption treatments. The initial adsorption pH value was adjusted by adding 0.1 mol L^−1^ hydrochloric acid or sodium hydroxide solution. The adsorbents (0.1 g) were added into heavy metal ion solution at different pH (2.0–8.0) for 24 h at 30 °C. Effect of contact time was conducted by adding 0.1 g of adsorbent into heavy metal solution (pH was about 7) at temperatures of 20 °C, 30 °C, and 40 °C. The supernatants were collected at 8 different time points within 24 h to analyze heavy metal concentrations, and the associated variations in concentration data were fitted to different kinetic models. For the thermodynamics adsorption study, the adsorbents (0.1 g) were added into 100 mL of individual heavy metal ion solutions with concentrations ranging from 25 to 200 mg L^−1^ at different temperatures (20 °C, 30 °C, and 40 °C) for 24 h. The adsorption isotherm data were fitted to different thermodynamics models.

The amount of heavy metal ions adsorbed to adsorbents was calculated using the following equation:

(1) $$q_{e}=\frac{\left(C_{0}-C_{e}\right)V}{m}$$

where *q*_e_ is the adsorption capacity (mg g^−1^) of the adsorbents at adsorption equilibrium, *C*_0_ and *C*_e_ are the initial and equilibrium concentration of the ions (mg L^−1^), respectively, *V* is the initial volume of solution (L), and *m* is the weight of the adsorbents (g).

## 3 Results and discussion

### 3.1 XRD analysis

**Figure 1:**
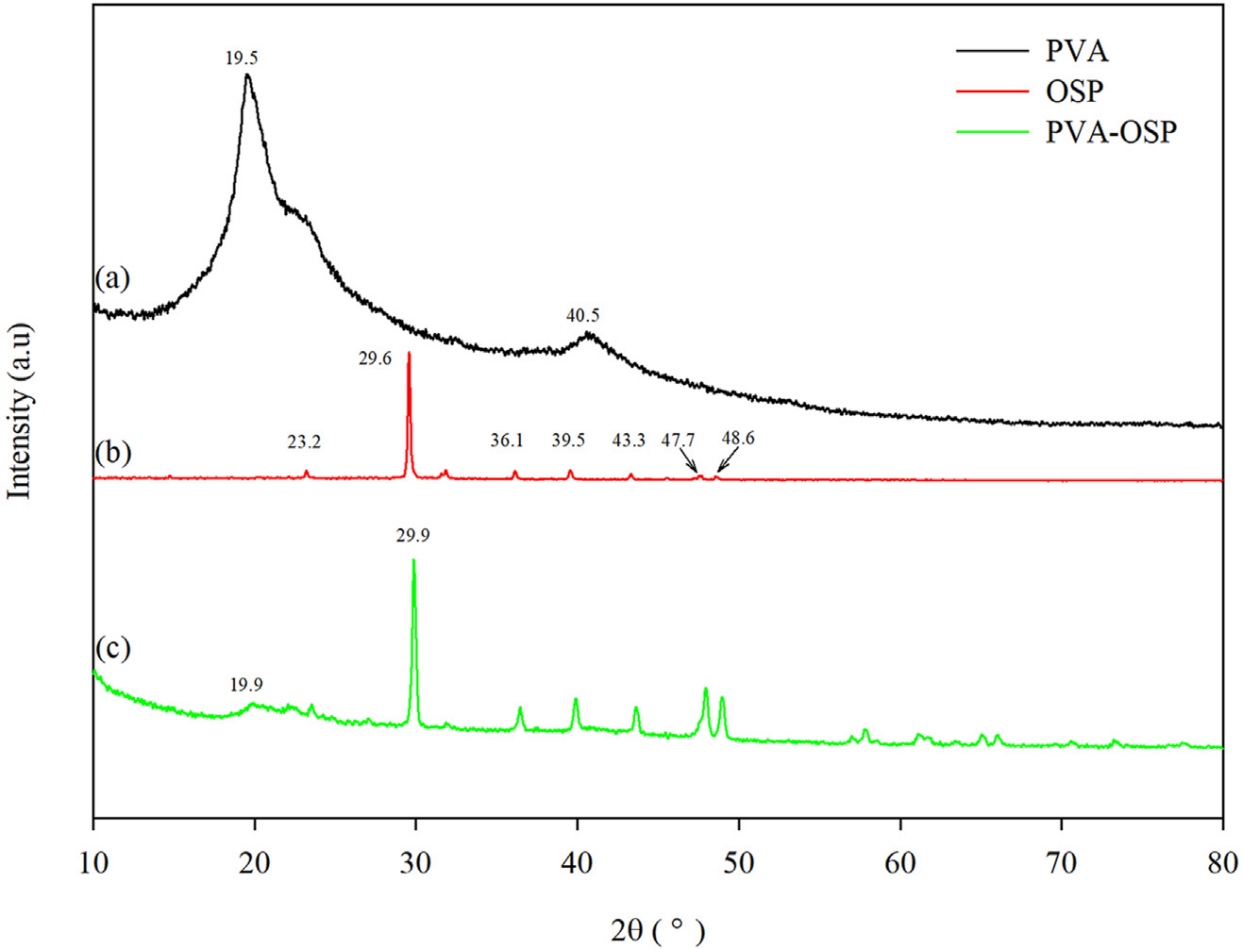
XRD patterns of (a) pure PVA, (b) OSP and (c) PVA-OSP.

XRD patterns are performed on the materials of PVA, OSP, and PVA-OSP, respectively. Pure PVA exhibits characteristic crystalline peaks at 2θ values of 19.5°, and 40.5°, which are related to the semi-crystalline nature of PVA (Figure 1a). The semi-crystalline structure is attributed to the intra-molecular and inter-molecular hydrogen bonds created by the individual monomer unit or even in the different monomer units (Aziz et al., 2017; Mallakpour and Khadem, 2017b). Figure 1b presents the strong diffraction peaks of OSP centered at 23.2°, 29.6°, 36.1°, 39.5°, 43.3°, 47.7°, and 48.6° assigned to the (012), (104), (110), (113), (202), (018) and (116) crystallographic planes of calcite, respectively. The result is in good agreement with reported data in the literature for calcite (Luo et al., 2020; Molki et al., 2019; Zhao et al., 2016).

The XRD spectrum indicates that the crystal form of the CaCO_3_ in the PVA-OSP material is highly similar to the standard card of calcite. The insertion of the OSP into the PVA matrix provided a few changes in the XRD pattern of PVA. A peak at 29.9° was observed as a result of the presence of OSP (Figure 1c). The peak area at 19.9° shrank and weakened significantly related to the reduction of the hydroxyl group in PVA, which implied that the reaction was likely to happen to the hydroxyl groups of PVA.

### 3.2 FI-RT analysis

**Figure 2:**
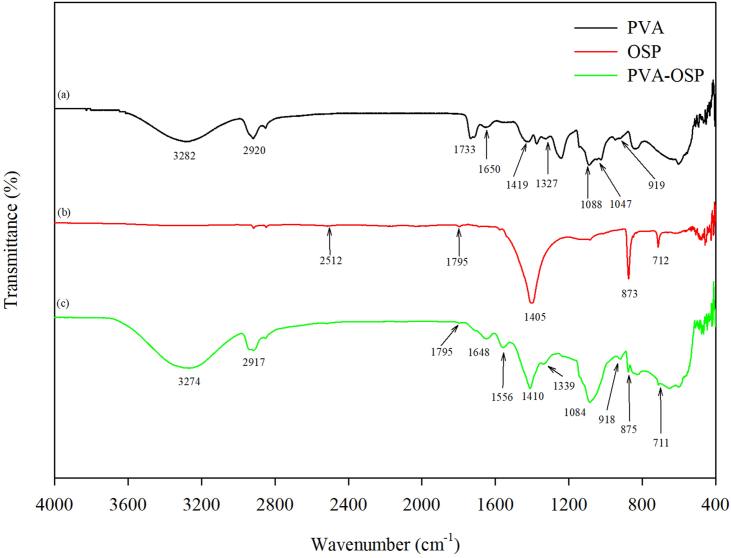
FT-IR spectra of (a) pure PVA, (b) OSP and (c) PVA-OSP.

For the pure PVA, a broad and intense band around 3282 cm^−1^ is observed, which is associated with the existence of a hydroxyl group (Raghu et al., 2005, 2009) from intra- and inter-molecular hydrogen bonding in the PVA chain (Figure 2a). Figure 2a also shows the appearances of the peaks at 1327, 1419, 1650, 1733, and 2920 cm^−1^, which are due to the C–H wagging vibration of CH_2_, C–H bending vibration of CH_2_, C=C stretching vibration of remaining acetate group, C=O stretching vibration of remaining acetate group, and C–H stretching vibration of CH_2_, respectively (Raghu et al., 2005, 2009; Rosli et al., 2021). The characteristic C–O stretching vibration of PVA is located at 1088 cm^−1^. The faint shoulder at 1047 cm^−1^ and weak peak at 919 cm^−1^ correspond to the crystalline domains and C–C stretching vibrations (Zheng et al., 2008; Rosli et al., 2021).

Figure 2b shows that FT-IR spectra of the OSP centered at 712, 873, and 1405 cm^−1^ are observed, corresponding to, respectively, the *ν*_4_ (in-plane bending), *ν*_2_ (out-of-plane bending), and *ν*_3_ (asymmetric stretching) vibration modes of CO_3_^2−^ in the calcite form. Besides, the characteristic of overlapped peaks around 1795, 2512, and 2874 are also observed (Zhao et al., 2016; Zhu et al., 2021).

Figure 2c displays the FT-IR characteristic of the PVA-OSP. A red shift of the hydroxyl group from 3282 cm^−1^ of pure PVA to 3274 cm^−1^ of the composite is found, resulting from the attendance of residual OH (Figure 2c). The PVA-OSP has a peak at 918 cm^−1^ relating to C–C stretching vibration but does not have crystalline domains. In contrast to the pure PVA, shifts of C–O stretching vibration (1088 cm^−1^ *vs.* 1084 cm^−1^), C–H wagging vibration (1327 cm^−1^ of pure PVA *vs.* 1339 cm^−1^ of PVA-OSP, hereafter), C–H bending vibration (1419 cm^−1^ *vs.* 1410 cm^−1^), C=C stretching vibration (1650 cm^−1^ *vs.* 1648 cm^−1^), C=O stretching vibration (1733 cm^−1^ *vs.* none) and C–H stretching vibration (2920 cm^−1^ *vs.* 2917) are observed. In contrast to the OSP, in-plane bending vibration of CO_3_^2−^ (712 cm^−1^ of OSP *vs.* 711 cm^−1^ of PVA-OSP, hereafter), out-of-plane bending vibration (873 cm^−1^ *vs.* 875 cm^−1^), asymmetric stretching vibration (1405 cm^−1^ *vs.* 1410 cm^−1^), and overlapped peak (1795 cm^−1^ *vs.* 1795 cm^−1^, 2512 cm^−1^ *vs.* none, 2874 cm^−1^ *vs.* none) are shown. In addition, some new peaks appeared (Figure 2c). A new adsorption band from 941 to 1226 cm^−1^ represents possible Si–O–Ca/Si–O–C vibration overlapped with specific bands of SiO_2_ focusing on the Si–O–Si, Si–O, and Si–OH vibrations (Ganie et al., 2020; Mallakpour and Khadem, 2017b; Rosli et al., 2021). The peak at 1556 cm^−1^ is associated with the stretching vibrations of –COO^−^. These results showed the occurrence of molecular interactions between organic and inorganic components in the preparation of the PVA-OSP composite.

### 3.3 TG-DSC analysis

**Figure 3:**
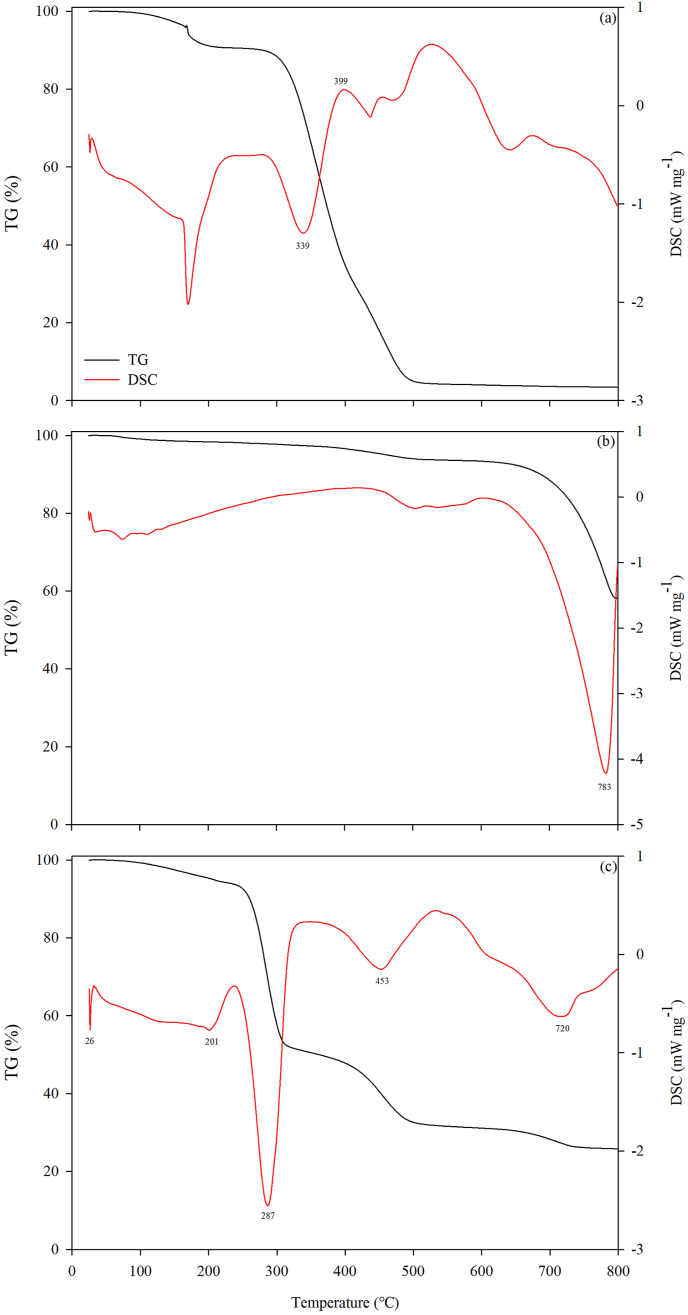
TG (black) and DSC (red) curves of (a) pure PVA, (b) OSP and (c) PVA-OSP.

The TG and DSC curves of the pure PVA, OSP, and PVA-OAP are illustrated in Figure 3. The thermosgram of the pure PVA displays three obvious decomposition stages (Figure 3a). The first stage at a temperature of 25–168 °C is due to the chemically or physically adsorbed hydrolyzed water molecules. The second decomposition stage is observed in the temperature range between 168 and 314 °C corresponding to the dissociation of inter- and intra-molecular hydrogen bonding and chain degradation. The last step of decomposition appears at around 314–500 °C attributing to the decomposition of the polymer backbone or decomposition of yielding carbon, polymer structure, and hydrocarbon residual in PVA (Reddy et al., 2019; Zanela et al., 2019). The incomplete melting endothermic peak at 339 °C was associated with a complicated process. The partial melting endothermic process of the PVA was covered by the subsequently fast exothermic decomposition process. In addition, there was an exothermic peak at 399 °C which might be due to the pyrolytic reaction and carbonization of PVA (Wei et al., 2018).

For the OSP, the weight loss from 25 to 642 °C was attributed to the thermal decomposition of organic substrates in the OSP. It can be seen that a rapid and large weight loss occurred at the temperature range from 642 to 797 °C, which is assigned to the thermal decomposition of CaCO_3_ (Figure 3b). A strong endothermic peak occurs at 724 °C, further confirming that a large amount of heat was absorbed by the decomposition of the CaCO_3_ (Zhao et al., 2016).

The TG-DSC curve of PVA-OSP exhibits four major decomposition stages of mass loss from the material over the 25–800 °C temperature range (Figure 3c). The first stage of weight loss happened below 240 °C, where the mass loss resulted from the loss of moisture and bounded volatile disintegrated products. An endothermic peak is observed at around 26 °C, which is likely related to the absorption of water between molecules within the composite. A minor endothermic peak appeared at 201 °C. The second stage of weight loss occurred between 240 and 302 °C, which was attributed to the cleavage of the polymer (e.g., inter- and intra-molecular hydrogen bonds, C–O–C bonds). The endothermic peaks at 287 °C verified the cleavage of these bonds within the material. The third stage appeared at a temperature of 302–497 °C. In this stage, the C–C bonds of the molecules within the materials were broken, producing carbon ash and high-boiling hydrocarbons. The endothermic peak at 453 °C indicated that the heat was absorbed to break the bonds in the backbone of the polymer chains. In the fourth stage (497–734 °C), the CaCO_3_ in the composite produced the decomposition reaction. The endothermic peak at 720 °C corresponded to the decomposition of the CaCO_3_ in large quantities. At the end of heating, there was 25.83% of the residue remained.

The results of the TG-DSC analysis suggest that the PVA-OSP composite had good thermal stability below 200 °C. The prepared composite can be used in conventional adsorption processes.

### 3.4 SEM analysis

**Figure 4:**
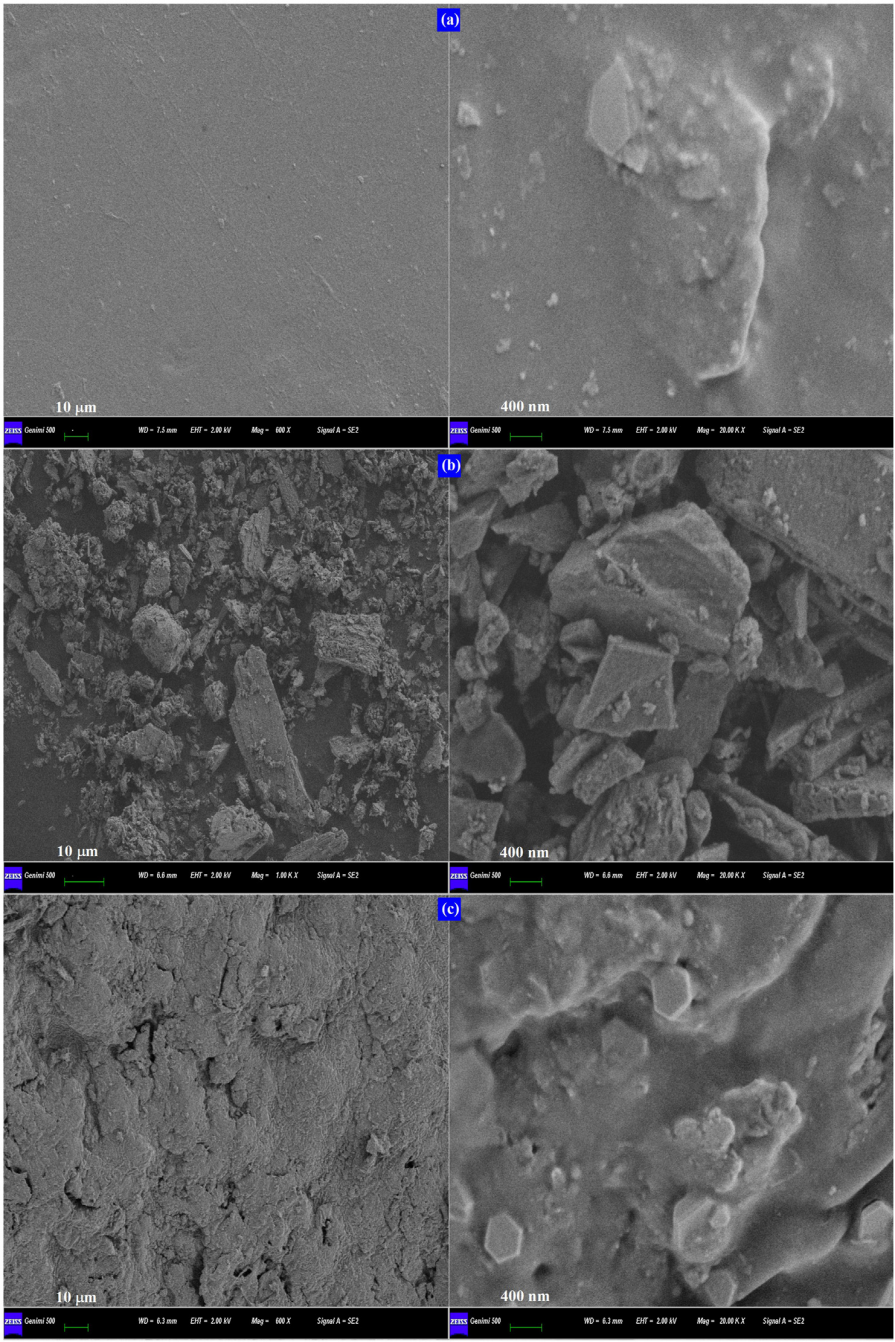
The SEM micrographs of (a) pure PVA, (b) OSP and (c) PVA-OSP.

The surface morphology of the pure PVA, OSP, and PVA-OSP was investigated by SEM micrographs. The micrograph of Figure 4a shows that the pure PVA exhibits a relatively smooth and uniform shape surface structure (Zhang et al., 2015). According to previous studies, the pure PVA membrane has a porous structure with average pore diameters ranging from 9 to 20 nm (Ge et al., 2021; Ghobashy, 2018).

The structure of natural oyster shells is mainly composed of a huge amount of prismatic layers with micro-pore sizes of 2–10 μm (Xia et al., 2021). Detailed structures of the OSP are observed through SEM micrographs on cross-sectional surfaces (Figure 4b). The surface of the grated OSP consisted of obvious plates, which formed a layered porous structure. The spatial structure creates a strong adsorption capacity (Huh et al., 2016; Khirul et al., 2019; Paradelo et al., 2016; Pu et al., 2019; Song and Gao, 2013; Xia et al., 2021). The physical and/or chemical cross-linking of PVA with PVA or other materials leads to the spontaneous formation of hydrogel with various porous structures (Ghobashy, 2018). The SEM image (Figure 4c) indicates apparent compatibility between flexible organic and rigid inorganic components. The PVA and silicon cross-linking agent formed a network structure, which was uniformly coated on the surface of the OSP. Compared to the pure PVA, slightly uneven areas and small crevices could be observed corresponding to weak attachment and poor miscibility between the organic and inorganic materials. The SEM micrographs of the composite provide direct evidence for decreased crystallinity as indicated in XRD analysis. It seems that PVA and silicon cross-linking agent act as adhesive to bond inorganic particles together, which could be confirmed by the FT-IR analysis above. The molecular interactions among hydroxyl in PVA, Si in cross-linking agent, Ca^2+^ in OSP, and water made more free water in the system and less bound water, further improving the pore structure in the composite (Xia et al., 2021).

### 3.5 Adsorption properties

#### 3.5.1 Effect of pH

The pH of the solution has a significant influence on the adsorption of heavy metal ions, which might affect surface charge distribution and ionization degree of the adsorbent, as well as the solubility and the existence form of adsorbate ion (Verma et al., 2008).

**Figure 5:**
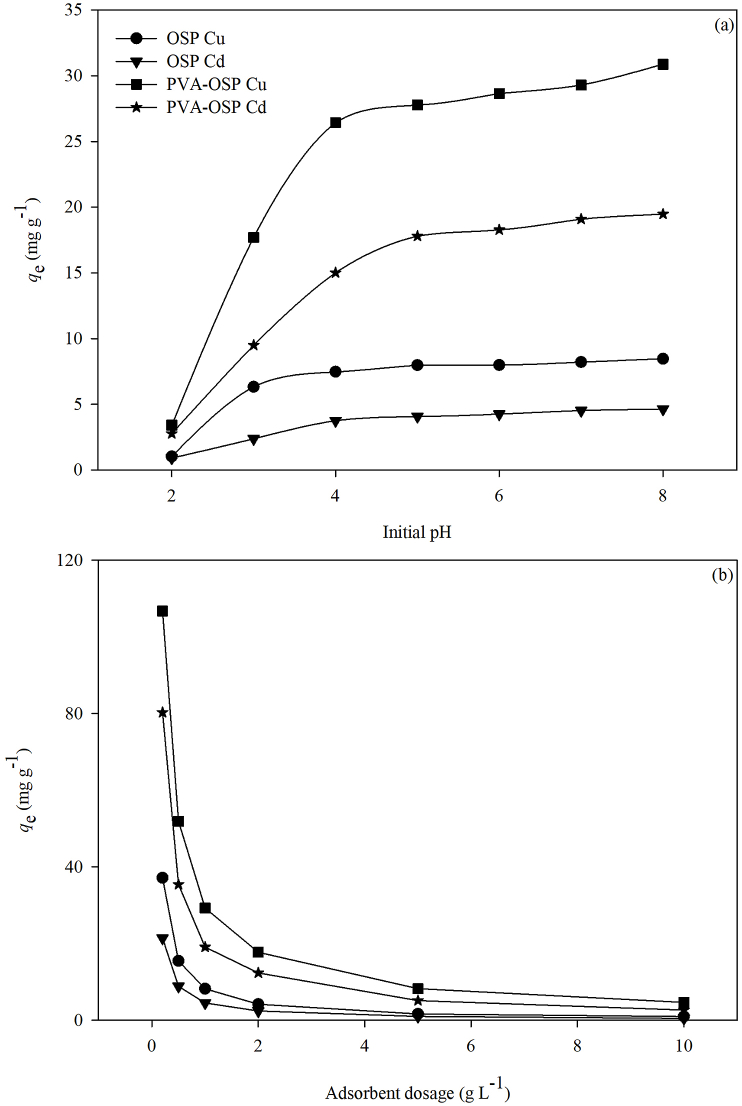
Effect of initial pH value (a) and adsorbent amount (b) on adsorption capacities of OSP and PVA-OSP to heavy metals.

When the pH value increased from 2 to 8, the removal efficiency of both OSP and PVA-OSP on Cu^2+^ and Cd^2+^ gradually increased and became stable when the pH reached 8 (Table 1; Figure 5a). The removal efficiencies of copper and cadmium by OSP were increased by 8.18 and 5.07 times, respectively, and the removal efficiencies by PVA-OSP were increased by 9.05 and 7.02 times, respectively. Calcium carbonate decomposes in acidic media. When the pH value is low, there is a large amount of H^+^ in the solution, which competes with heavy metal ions for CO_3_^2−^ released from the surface of OSP. The formation of carbonate precipitation on the adsorbent surface is thereby prevented. As the pH value increases, the H^+^ concentration in the solution decreases, and heavy metal ions can obtain more CO_3_^2−^ to form carbonate precipitation, increasing the adsorption capacity. At the same time, the negative charge on the adsorbent surface also increases with the increase of pH, which weakens the electrostatic repulsion between the adsorbent surface and metal cations. This process also contributes to the increase of adsorption capacity. With the increase of pH value, metal cations gradually transform into the hydroxide structure, decreasing soluble cation concentration. In addition, the strong coordination interactions between metal cations and functional groups in the PVA-OSP, such as –OH and C=O, can increase the removal efficiency of heavy metals.

**Table 1:** Results of two-way analyses of variance (ANOVA) on the effects of adsorbent material and initial pH value as well as adsorbent material and addition amounts of adsorbent on adsorption capacity *q*_e_ to Cu^2+^ and Cd^2+^.

Initial pH				Addition amount		
Adsorbate	Material (M)	pH	M × pH	Material (M)	Amount (A)	M × A
Cu^2+^	895.598∗∗∗	70.934∗∗∗	24.236∗∗∗	409.619∗∗∗	293.681∗∗∗	66.096∗∗∗
Cd^2+^	994.848∗∗∗	67.355∗∗∗	27.651∗∗∗	495.994∗∗∗	302.950∗∗∗	99.328∗∗∗

*F* values and significance levels (∗∗∗*P* < 0.001, ^n.s.^*P* ≥ 0.05) are given.

#### 3.5.2 Effect of adsorbent amount

The amount of adsorbent can directly affect the treatment effect of water quality and cost, which is an important factor in practical application. In Figure 5b, it is clearly shown that higher adsorbent dosages would result in more heavy metal ions removal from solution when the dosage increased from 0.2 to 10 g L^−1^, but smaller adsorption capacity *q*_e_. However, the increasing trend of heavy metal ions removal gradually becomes less obvious when the dosage is higher than 2 g L^−1^ (Table 1).

#### 3.5.3 Adsorption kinetic

The adsorption kinetics is critical to determine the rate of heavy metals adsorption by PVA-OSP at different time intervals and to understand the associated adsorption mechanism. The kinetics of adsorption behaviors of heavy metals onto the PVA-OSP were analyzed by pseudo-first-order kinetics (Eq. (2)), pseudo-second-order kinetics (Eq. (3)), and intraparticle diffusion models (Eq. (4)):

(2) $${q_{t}=q_{e}(1-e}^{-k_{1}t})$$

(3) $$q_{t}=\frac{k_{2}tq_{e}^{2}}{1+k_{2}tq_{e}}$$

(4) $$q_{t}=k_{i}t^{\frac{1}{2}}+c$$

where *q*_e_ and *q*_t_ represent the amount of heavy metal ions adsorbed (mg g^−1^) at equilibrium and time t (min), respectively; *k*_1_ (min^−1^), *k*_2_ (g mg^−1^ min^−1^) and *k*i ($mg\;g^{-1}\;\min^{\frac{1}{2}}$) refer to the pseudo-first-order, pseudo-second-order, and intraparticle diffusion rate constants, respectively; and *c* is the intercept (Xu et al., 2019; Zhang et al., 2021). Adsorption kinetic curves were made by taking adsorption time as abscissa and *q*_e_ as ordinate. Sigmaplot (Systat Software, Inc., Erkrath, Germany) was used for plotting and curve fitting to obtain each parameter.

**Figure 6:**
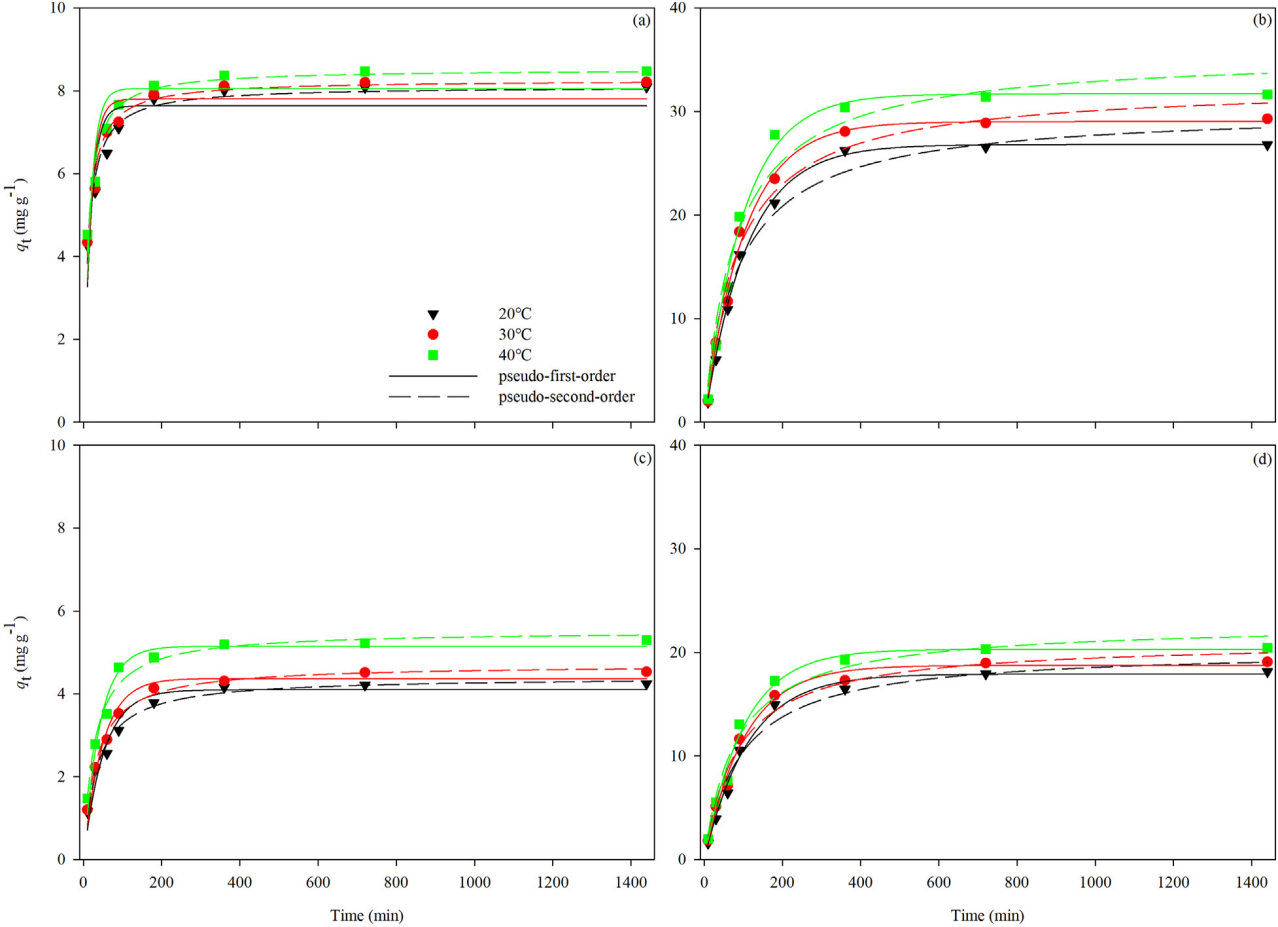
Adsorption kinetics of Cu^2+^ (a, b) and Cd^2+^ (c, d) by OSP (a, c) and PVA-OSP (b, d) and the fitting curves of pseudo-first-order model (solid line) and pseudo-second-order model (dashed line) at 20 °C (black), 30 °C (red), and 40 °C (green).

**Figure 7:**
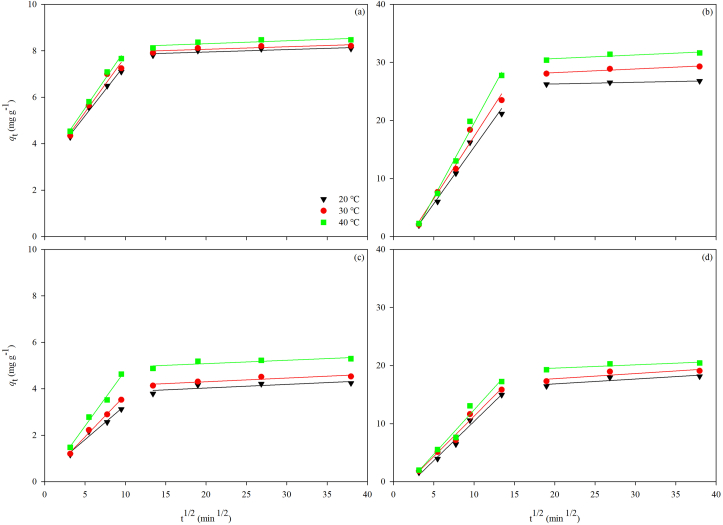
Adsorption kinetics of Cu^2+^ (a, b) and Cd^2+^ (c, d) by OSP (a, c) and PVA-OSP (b, d) and the fitting curves of intraparticle diffusion model at 20 °C (black), 30 °C (red), and 40 °C (green).

The PVA-OSP has a stronger adsorption capacity for heavy metals than the OSP (Table 2; Figures 6 and 7). At the same temperature, the *q*_e_ of the OSP increases rapidly in the first 180 min (Table 2; Figures 6a, 6c, 7a and 7c)., while the PVA-OSP increases faster in the first 360 min (Table 2; Figures 6b, 6d, 7b and 7d). As observed in Figure 6 and Table 3, the correlation coefficients (*R*^2^) of the pseudo-second-order model fits of Cu^2+^ and Cd^2+^ adsorption onto OSP were higher than the corresponding coefficients from the fits by the pseudo-first-order kinetic model. The *q*_e, cal_ (mg g^−1^) values obtained from the pseudo-second-order kinetic model were closer to the *q*_e, exp_ (mg g^−1^) values in Table 3. In the pseudo-first-order and pseudo-second-order models, larger rate constants (*K*) indicate a faster rate of adsorption of the heavy metal ions compared to smaller values. The adsorption rate of Cu^2+^ was faster than the adsorption rate of Cd^2+^. Thus, the adsorption process suggests that the adsorption of Cu^2+^ and Cd^2+^ by the OSP can be rationally assessed by the pseudo-second-order model. In contrast, the correlation coefficients of the PVA-OSP display close evaluated *q*_e, exp_ (mg g^−1^) values and good fitness with the experimental *q*_e_ value from the pseudo-first-order kinetic model than the second-order equation. The adsorption of Cu^2+^ by the PVA-OSP was still faster than the adsorption of Cd^2+^ according to the experimental data (Figure 6).

**Table 2:** Results of three-way analyses of variance (ANOVA) on the effects of adsorbent material, temperature and adsorption time as well as adsorbent material, temperature and initial concentration of adsorbates on adsorption capacity *q*_e_ to Cu^2+^ and Cd^2+^.

Adsorption kinetics							
Adsorbate	Material	Temperature (Te)	Time (Ti)	M × Te	M × Ti	Te × Ti	M × Te × Ti
Cu^2+^	1891.062∗∗∗	17.777∗∗∗	254.660∗∗∗	11.334∗∗∗	152.152∗∗∗	0.705^n.s.^	0.691^n.s.^
Cd^2+^	2051.010∗∗∗	19.554∗∗∗	226.165∗∗∗	2.994^n.s.^	110.160∗∗∗	0.402^n.s.^	0.119^n.s.^

Adsorption isotherm							
Adsorbate	Material	Temperature (Te)	Initial concentration (IC)	M × Te	M × IC	Te × IC	M × Te × IC
Cu^2+^	2070.746∗∗∗	8.830∗∗∗	83.986∗∗∗	6.176∗∗	57.824∗∗∗	0.251^n.s.^	0.213^n.s.^
Cd^2+^	2326.789∗∗∗	7.782∗∗∗	80.623∗∗∗	2.876^n.s.^	58.402∗∗∗	0.149^n.s.^	0.110^n.s.^

*F* values and significance levels (∗∗∗*P* < 0.001, ∗∗*P* < 0.01, ^n.s.^*P* ≥ 0.05) are given.

**Table 3:** Kinetic parameters of the pseudo-first-order, pseudo-second-order kinetic models for the adsorption of Cu^2+^ and Cd^2+^by the OSP and the PVA-OSP.

Material	Adsorbate	*T* (°*C*)	*q*_e, exp_ (mg g^−1^)	Pseudo-first-order model			Pseudo-second-order model		
				*q*_e, cal_ (mg g^−1^)	*k*_1_ (min^−1^)	R^2^	*q*_e, cal_ (mg g^−1^)	*k*_1_ (g mg^−1^ min^−1^)	R^2^
OSP	Cu^2+^	20	8.0914	7.6365	0.0559	0.7795	8.1059	0.0111	0.9623
		30	8.2132	7.8048	0.0570	0.8229	8.2622	0.0113	0.9739
		40	8.4753	8.0570	0.0569	0.8112	8.5232	0.0110	0.9696
	Cd^2+^	20	4.2433	4.1002	0.0192	0.9429	4.4096	0.0066	0.9857
		30	4.5312	4.3718	0.0207	0.9685	4.7080	0.0066	0.9936
		40	5.2954	5.1499	0.0238	0.9706	5.5220	0.0066	0.9794
PVA-OSP	Cu^2+^	20	26.7911	26.8077	0.0092	0.9965	30.2080	0.0004	0.9788
		30	29.2965	29.0292	0.0097	0.9944	32.6203	0.0004	0.9800
		40	31.8107	31.7061	0.0100	0.9939	35.6160	0.0003	0.9686
	Cd^2+^	20	18.1439	17.9165	0.0089	0.9918	20.3216	0.0005	0.9762
		30	19.0869	18.7595	0.0097	0.9906	21.1174	0.0006	0.9798
		40	20.4398	20.3527	0.0101	0.9886	22.8263	0.0005	0.9680

As shown in Figure 7, the adsorption of both Cu^2+^ and Cd^2+^ by the OSP and the PVA-OSP seemed to involve different steps. The relevant parameters of the intraparticle diffusion model are listed in Table 4. For the OSP, the heavy metal ions reacted quickly with the CaCO_3_ on the surface of the adsorbent in the first stage (Figure 7a and 7c). Due to the precipitation and stack of the OSP, CaCO_3_ might not be fully utilized, which resulted in a much smaller adsorption rate represented by *k*_i_ values in the second stage (Table 4 and Figure 7). For the PVA-OSP, the solution first entered the hydrogels pore structure, and then the heavy metal ions came to CaCO_3_ on the surface of the adsorbent in the first stage (Figure 7b and 7d). The existence of hydroxyl allowed intermolecular and hydrogen bonds within the ion, resulting in good water absorption and retention of hydrogel. In the second stage, the calculated *k*_i, 2_ values were much smaller than the *k*_i,1_ values, indicating that the reaction products diffused into the interior of the adsorbent after the heavy metal ions reacted with the superficial CaCO_3_. Because of the closely interconnected PVA-OSP molecules, increase in resistance of the heavy metal ions to internal diffusion and decrease in the adsorption rate over time could be observed.

**Table 4:** Intraparticle diffusion model parameters for the adsorption of Cu^2+^ and Cd^2+^ by the OSP and the PVA-OSP.

Material	Adsorbate	*T* (°*C*)	Intraparticle diffusion model					
			*k*_i, 1_ (mg g^−1^ min^1/2^)	*c* (mg g^−1^)	R^2^	*k*_i, 2_ (mg g^−1^ min^1/2^)	*c* (mg g^−1^)	R^2^
OSP	Cu^2+^	20	0.4440	2.9840	0.9898	0.0105	7.7389	0.7049
		30	0.4794	2.9600	0.9636	0.0109	7.8486	0.7077
		40	0.5055	3.0040	0.9910	0.0127	8.0541	0.6936
	Cd^2+^	20	0.2970	0.3280	0.9726	0.0157	3.7202	0.6215
		30	0.3609	0.1288	0.9932	0.0160	3.9849	0.8260
		40	0.4794	0.0017	0.9865	0.0145	4.7959	0.6963
PVA-OSP	Cu^2+^	20	1.9480	-4.0687	0.9812	0.0287	25.7231	0.9719
		30	2.1459	-4.2100	0.9757	0.0635	26.9740	0.9170
		40	2.5636	-6.0806	0.9908	0.0628	29.4032	0.8151
	Cd^2+^	20	1.3517	-3.1295	0.9835	0.0841	15.1668	0.7621
		30	1.3931	-2.6278	0.9822	0.0887	15.9753	0.7176
		40	1.5322	-2.9484	0.9729	0.0572	18.4180	0.7512

#### 3.5.4 Adsorption isotherm

The adsorption isotherm data could describe adsorption capacity, surface properties, and affinity of the adsorbents, and help to understand the mechanism of heavy metal ions adsorption onto the adsorbents. The experimental data were examined by Langmuir, Freundlich, and Temkin models for the OSP and PVA-OSP composite. The Langmuir model, shown in Eq. (5), is a homogeneous monolayer adsorption on the surface with identical sites (El Atouani et al., 2019; Homagai et al., 2022; Kim and Singh, 2022).

(5) $$q_{e}=\frac{q_{m}K_{L}C_{e}}{1+K_{L}C_{e}}$$

where *q*_e_ is the amount of heavy metal ions adsorbed by adsorbent at equilibrium (mg g^−1^) which could be obtained with Eq. (1), *K*_L_ is the Langmuir adsorption constant (L mg^−1^), *q*_m_ is the maximum amount of adsorption corresponding to complete monolayer coverage on the surface (mg g^−1^), and *C*_e_ is the equilibrium concentration of heavy metal ion solution (mg L^−1^).

The Freundlich model, shown in Eq. (6), describes an experiential model with heterogeneous multilayer adsorption on surfaces (El Atouani et al., 2019; Kim and Singh, 2022).

(6) $$q_{e}=K_{F}C_{e}^{\frac{1}{n}}$$

where *K*_F_ is the adsorption constant for the adsorption capacity (mg g^−1^), and $\frac{1}{n}$ is the adsorption intensity.

The Temkin model, shown in Eq. (7), considers the interaction force between the adsorbent and the adsorbate. It is assumed that this force causes the adsorption heat of all molecules adsorbed on the adsorbent surface to satisfy a linear decreasing relationship with its coverage. Adsorption is characterized by a uniform distribution of binding energy up to a certain maximum value (El Atouani et al., 2019; Kim and Singh, 2022).

(7) $$q_{e}=\frac{RT}{b_{T}}\ln\;K_{T}+\frac{RT}{b_{T}}\ln\;C_{e}$$

where *T* is the absolute solution temperature (K), *R* is the universal gas constant (8.314 J mol^−1^ K^−1^), and *K*_T_ (L mol^−1^) is the Temkin equilibrium binding constant corresponding to the maximum binding energy. The variation of adsorption energy *b*_T_ (J mol^−1^) is a constant related to the heat of the adsorption process, where the positive or negative value of *b*_T_ shows that the adsorption process is an endothermic or exothermic process, respectively.

**Figure 8:**
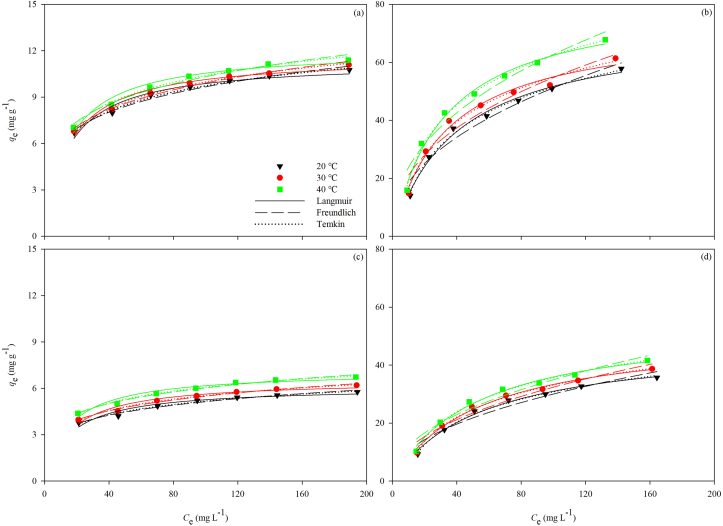
Adsorption isotherms of Cu^2+^ (a, b) and Cd^2+^ (c, d) by OSP (a, c) and PVA-OSP (b, d) and the fitting curves of Langmuir (solid line), Freundlich (dashed line) and Temkin (dot line) models at 20 °C (black), 30 °C (red), and 40 °C (green).

Adsorption isotherms curves were made by taking *C*_e_ as abscissa and *q*_e_ as ordinate. Sigmaplot was also used for plotting and curve fitting to obtain each parameter. With the increase of temperature, the adsorption capacity of the OSP (Figure 8a and 8c) and the PVA-OSP (Figure 8b and 8d) to heavy metals also increased (Tables 1 and 2). The relevant thermodynamic parameters obtained from fitting the isotherm data by the Langmuir, Freundlich, and Temkin models are listed in Table 5. For the OSP, the *R*^*2*^ of the thermodynamic data corresponding to the adsorption of Cu^2+^ and Cd^2+^ obtained from the Temkin model and the Freundlich model were higher than the *R*^*2*^ values from the Langmuir model. For the PVA-OSP, the Temkin model and the Langmuir model had larger *R*^*2*^ values acquired from the thermodynamic data than the Freundlich model. Particularly, the Temkin model fits the experimental data best (Figure 8).

**Table 5:** Langmuir, Freundlich and Temkin isotherm constants for the adsorption of Cu^2+^ and Cd^2+^ by the OSP and the PVA-OSP.

Material	Adsorbate	*T* (°*C*)	Langmuir model			Freundlich model			Temkin model		
			*q*_m_ (mg g^−1^)	*K*_L_ (L mg^−1^)	*R*^*2*^	*K*_F_ (mg g^−1^)	$\frac{1}{n}$	*R*^*2*^	*K*_T_ (L g^−1^)	*b*_T_ (J mol^−1^)	*R*^*2*^
OSP	Cu^2+^	20	11.3029	0.0695	0.9621	3.7952	0.2031	0.9844	2.2382	1354.4035	0.9928
		30	11.6377	0.0676	0.9685	3.8221	0.2070	0.9856	2.0318	1340.9178	0.9952
		40	12.0876	0.0687	0.9701	4.0123	0.2052	0.9796	2.1101	1340.1601	0.9916
	Cd^2+^	20	6.0891	0.0636	0.9325	2.0095	0.2040	0.9774	2.0839	2513.4053	0.9785
		30	6.5273	0.0626	0.9457	2.1179	0.2072	0.9868	1.9382	2397.1743	0.9886
		40	7.1014	0.0663	0.9403	2.3903	0.2011	0.9852	2.2728	2325.6178	0.9867
PVA-OSP	Cu^2+^	20	72.5603	0.0248	0.9895	6.7034	0.4416	0.9624	0.2206	146.4499	0.9951
		30	77.8993	0.0262	0.9836	7.3787	0.4404	0.9495	0.2235	139.0744	0.9908
		40	82.6117	0.0309	0.9902	9.0032	0.4215	0.9590	0.2778	138.5379	0.9958
	Cd^2+^	20	48.1937	0.0180	0.9927	3.5862	0.4617	0.9493	0.1503	214.3409	0.9951
		30	51.4107	0.0185	0.9923	3.8536	0.4625	0.9552	0.1559	208.7555	0.9963
		40	54.6958	0.0191	0.9902	4.1925	0.4607	0.9532	0.1611	202.5320	0.9949

#### 3.5.5 Adsorption mechanism

The adsorption process of Cu^2+^ and Cd^2+^ by the OSP can be better represented by the pseudo-second-order kinetic model. What occurred between the adsorbent and the heavy metal ions was a rate-limited chemisorption process, which involved the interaction between the adsorbent and the adsorbate, sharing or substitution of electrons. However, when using the OSP solely for adsorption, the powders were liable to stack together and precipitate, thus hindering the exchange process and resulting in the relatively low adsorbing capacity for Cu^2+^ and Cd^2+^ (Pu et al., 2019; Xu et al., 2019). The adsorption isotherm process of the OSP is well-fitted with the Freundlich model and Temkin model (Figure 8a and 8c). Multilayer adsorption of heavy metal ions occurred on the uneven surface of the OSP. In the Freundlich model, the magnitude of the exponent, $\frac{1}{n}$, reveals the favorability of adsorption. It is relatively easy for an adsorbent to adsorb adsorbate when $\frac{1}{n}$ is less than 1. In Table 5, all $\frac{1}{n}$ values are less than 1, which indicates that the OSP is good at adsorbing Cu^2+^ and Cd^2+^ (El Atouani et al., 2019; Mboyi et al., 2021).

While in the PVA-OSP composite, the adsorption process exhibited completely different characteristics (Table 5; Figure 8b and 8c). The pseudo-first-order kinetics model and the Langmuir model are best-suited for fitting the experimental data. In theory, the good dispersion of the OSP and the good hydrophilicity of hydrate dramatically increased the meeting opportunity of Ca^2+^ and target ions (Pu et al., 2019), ultimately increasing the Cu^2+^ and Cd^2+^ adsorption of the porous composite. In other words, it is assumed that adsorbate firstly diffused from the solution to the solid surface of the PVA-OSP composite, then entered porous composite with the solution due to the strong hydroscopicity of the composite, and came to the OSP in the composite. The pseudo-first-order kinetic model is based on the relationship between reactant concentration and reaction rate. Experimental data have shown that the application of the pseudo-first-order kinetic model is successful when the adsorption process occurs rapidly with the PVA-OSP. During the adsorption process, the adsorption was likely to be controlled by the diffusion process, and the difference between the adsorption concentration at a certain moment and the adsorption concentration at the equilibrium moment determined the adsorption rate at this moment, meaning the external mass transfer resistance was the limiting factor of the process (Zhang et al., 2021). The adsorption rate was linearly proportional to the remaining amount of adsorption and associated retention. The Langmuir model is based on homogeneous monolayer adsorption with all active sites having the same affinity for the adsorbate (El Atouani et al., 2019; Kim and Singh, 2022). From the SEM images, it can be seen that the PVA hydrate covered the surface of the OSP to form organic layers, which played an important role in adsorption (Figure 4).

The intraparticle diffusion model indicates that the diffusion process of each heavy metal ion adsorption has two distinct diffusion stages in both the OSP and the PVA-OSP (Figure 7). For both adsorbents, the heavy metal ions diffused rapidly on the adsorbents' surface in the first stage, then the diffusion rate decreased and tended to be stable (Figure 7). In the beginning, the chemical reaction should take place on the surface of the OSP, while both the physical and chemical adsorption should take place on the surface of the PVA-OSP (Pu et al., 2019; Zhang et al., 2021). During the adsorption increment process, intraparticle diffusion was the main controlling factor. Heavy metal ions slowly entered the internal structures of adsorbents by intraparticle diffusion and eventually remained in the adsorbents by chemical reaction. In the final equilibrium stage, the concentration of heavy metal ions in the solution decreased and the particle diffusion began to slow down to gradually reach adsorption equilibrium.

The Temkin isotherm model was generally used to determine the binding energies of the adsorption process. The positive value of the variation of adsorption energy *b*_T_ (J mol^−1^), a constant related to the heat of the adsorption process, indicates that the adsorption of different heavy metal ions by both the OSP and the PVA-OSP is an endothermic process (El Atouani et al., 2019). Since the *b*_T_ values are all less than 8 kJ mol^−1^, the interactions between the adsorbents and the adsorbates are weak, and therefore the adsorption mechanism here should be merely that of ion exchange (Ghogomu et al., 2013). The adsorption capacity of the adsorbents for heavy metal ions increased with the increase in temperature. The adsorbent was likely to expand when heated, which increased the contact area between the internal OSP and heavy metal ions, leading to an increase in the adsorption capacity of the adsorbates (Zhang et al., 2021).

## 4 Conclusions

The PVA-OSP porous composite with potential applications in the removal of heavy metal ions from wastewater was first prepared in this study. The structure and thermal analyses of the produced composite confirmed that not only was the occurrence of interactions between organic (PVA) and inorganic (Na_2_SiO_3_ and waste shell) components but also had good thermal stability. According to the results, the pseudo-first-order kinetic model as well as the Temkin and Langmuir isotherm model exhibited well fitness with the experimental data achieved from isotherm and kinetic studies with the PVA-OSP. Contrastingly, the pseudo-second-order kinetic model as well as the Temkin and Freundlich isotherm model fitted the isotherm and kinetic data of the OSP better. The adsorption capacities of Cu^2+^ and Cd^2+^ onto the PVA-OSP composite were about 6.64 and 7.83 times the adsorption capacities of the OSP on average, respectively, according to the fitting of the thermodynamic data by the Langmuir model. It indicates that the modified shell material can play a better role in adsorption. Good dispersion of OSP in the composite and the high hydrophilicity of PVA-OSP improve pore diffusion obstruction of heavy metals in the composite material. The composite material developed in this study, which ameliorates agglomeration and loss of powders, could be a good candidate material for the treatment of wastewater caused by heavy metal pollution. Based on the preparation method proposed in this paper, the optimization of the preparation method, composition ratio and adsorption selectivity of heavy metals can be further studied in the future.

It should be noted that this study had been primarily concerned with the feasibility of the PVA-OSP composite adsorption of heavy metals and did not include the optimization of the preparation method and composition ratio of this material. Besides, single Cu^2+^ and Cd^2+^ were respectively taken as the treatment objects. The actual wastewater generally contains a variety of heavy metal ions and other pollutants, which may form a complex system of competitive adsorption. To realize the practical application of the adsorbent, further research should be conducted on the removal of pollutants in the complex system.

## Declarations

### Author contribution statement

Zhenfeng Zhou: Conceived and designed the experiments; Contributed reagents, materials, analysis tools or data; Wrote the paper.

Yinuo Wang; Shu Sun; Yicheng Wang: Performed the experiments; Analyzed and interpreted the data; Wrote the paper.

Liang Xu: Conceived and designed the experiments; Analyzed and interpreted the data; Contributed reagents, materials, analysis tools or data; Wrote the paper.

### Funding statement

This work was supported by the Key 10.13039/100006190Research and Development Project of Ningxia Hui Autonomous Region [2021BEG03013], 10.13039/501100001809National Natural Science Foundation of China [41601339].

### Data availability statement

Data included in article/supp. material/referenced in article.

### Declaration of interest’s statement

The authors declare no conflict of interest.

### Additional information

No additional information is available for this paper.
